# Regulation of baby food marketing in Thailand: a NetCode analysis

**DOI:** 10.1017/S1368980022001446

**Published:** 2022-10

**Authors:** Nisachol Cetthakrikul, Matthew Kelly, Cathy Banwell, Phillip Baker, Julie Smith

**Affiliations:** 1 National Centre for Epidemiology and Population Health, Australian National University, Canberra, ACT 2601, Australia; 2 International Health Policy Program, Ministry of Public Health, Nonthaburi, Thailand; 3 Institute for Physical Activity and Nutrition, Deakin University, Geelong, Australia

**Keywords:** Baby food marketing, Violation, International Code of Marketing of Breast-milk Substitutes, NetCode, Thailand

## Abstract

**Objective:**

To report on the prevalence of different types of breast-milk substitutes (BMS) marketing and the compliance of such marketing with the ‘Control of Marketing of Infant and Young Child Food Act 2017’ (The Act) and the ‘International Code of Marketing of Breast-milk Substitutes (WHO Code)’ in Thailand.

**Design:**

Cross-sectional quantitative study, guided by the WHO/UNICEF NetCode Periodic Assessment Protocol.

**Setting:**

Health facilities and retail outlets in Bangkok, Thai media.

**Participants:**

Mothers of 0–2-year-old children, health professionals, promotions at retail outlets and health facilities, product labels, marketing on television and the internet.

**Results:**

Marketing to mothers was highly prevalent, mostly from electronic or digital media, while BMS companies provided items to health professionals to distribute to mothers. Promotional materials in health facilities displayed company brands or logos. At retail outlets, most promotions were price-related. Approximately two-fifths of labels contained nutrition or health claims. Television marketing was growing-up-milk (GUM) advertisements, while internet promotions were varied from price-related materials to product reviews. Most instances of non-compliant BMS marketing with the Act were advertisements to mothers, and most were infant formula. Most non-compliant BMS marketing with the WHO Code was mainly concerned GUM, which are not covered by the Act and appeared in the media.

**Conclusions:**

BMS marketing does not fully comply with the Act or the WHO Code. The Thai government should conduct regular monitoring and enforcement activities, educate health professionals, and strengthen the Act’s provisions on the media and GUM to fully align with the WHO Code.

The WHO recommends infants initiate breast-feeding in the first hour of life, continue to breastfeed exclusively for 6 months, and thereafter begin to be fed appropriate and safe complementary foods, while breast-feeding continues for up to 2 years of age and beyond^(1)^. These recommendations reflect evidence of the benefits of breast-feeding to the health and development of children, and for women’s health, in all countries^(2–12)^.

The promotion of breast-milk substitutes (BMS) by the baby food industry, including medical marketing to health professionals, direct to consumer advertising, and product strategies such as cross-promotion, is a key factor that undermines breast-feeding worldwide^(13)^. Exposure to such marketing results in increased bottle-feeding and reduced initiation, exclusivity and duration of breast-feeding irrespective of the country context. Such marketing may encourage mothers to hold negative attitudes towards breast-feeding^(14–17)^ and develop positive attitudes towards the use of commercial milk formulas^(18)^, and it reduces self-confidence in their ability to exclusively breastfeed^(19)^.

To protect mothers from BMS marketing, Member State governments at the World Health Assembly (WHA) adopted the International Code of Marketing of Breast-milk Substitutes Resolution WHA 34·22 in May 1981 and have since updated this through subsequent WHA Resolutions every 2 years (WHO Code)^(20)^. The WHO Code sits within a comprehensive package of policy actions outlined in the WHO/UNICEF Global Strategy on Infant and Young Child Feeding, and contains provisions to control BMS marketing to the public, mothers and health professionals, including in healthcare facilities, and on product labels^(20)^.

Thailand adopted provisions of the WHO Code without including penalties to control BMS marketing in 1981. Because of evidence from many countries that BMS companies were failing to comply with the WHO Code^(21,22)^, including reports of multiple Code violations in Thailand^(23)^, the WHA resolved in 2010 (WHA 63.23) that all Member States should legislate the WHO Code into national law, monitor company compliance and enforce the law when violations occur. In 2010, the 3rd National Health Assembly of the Thai government adopted a resolution to control the marketing of BMS through new laws^(24)^. The Department of Health advocated for enacting the WHO Code into law, and in 2017, the Control of Marketing Promotion of Infant and Young Child Food Act B.E. 2560 (the Act) came into effect.

In line with the WHO Code and subsequent WHA resolutions, and as Thailand is a party to the Convention on the Rights of the Child, the Act aims to control the marketing of BMS^(25)^ and protect the rights of all infants and young children by ensuring that parents are informed of the benefits of breast-feeding, without commercial influence on their decisions about feeding^(26)^. Its provisions address promotions to mothers and the public, at health facilities and to health professionals, scientific meetings, and the labelling of BMS^(27)^. The Act provisions follow the WHO Code because the WHO Code is intended as the minimum standard for national law. However, evidence suggests that the scope of provisions and strength of Thailand’s Act was significantly narrowed due to corporate political activity during the policy formulation^(28)^ and external pressure from other Member States in the WTO^(29)^.

Breast-feeding practices in Thailand remain far from WHO recommendations. In 2019, only 14 % of infants were exclusively breastfed to 6 months, and just 15 % of children aged 20–23 months were still breastfed^(30)^. The percentage of value growth of baby food marketing between 2015 and 2020 was 20·8. It increased from 2019 by 5·9 %. The BMS market in Thailand was 32 708·7 million baht (965·70 USD million) in 2020^(31,32)^. These trends are also reflected throughout developing countries of the Southeast Asia region, where commercial milk formula sales have grown rapidly in recent decades. This region is more important to the BMS industry, in terms of total market value than the US and European commercial milk formula markets combined^(13,33)^.

To understand the effectiveness of the Thai Act and inform actions to strengthen its provisions and enforcement, the prevalence and compliance of BMS marketing with Thai law and the WHO Code needs to be assessed. This study aims to provide a comprehensive and detailed report on the types and prevalence of BMS marketing in Thailand and on compliance of such marketing with the Act and with the WHO Code.

## Methods

This study employed the NetCode Periodic Assessment Protocol^(23)^, based on a cross-sectional quantitative design. The NetCode Protocol is part of a toolkit developed by the WHO and UNICEF to assist governments in establishing a sustainable system that will monitor, detect and report violations of national laws and the WHO Code^(23)^. The objectives of the NetCode Protocol include quantifying the level of compliance with national laws and the WHO Code and identifying gaps and issues that will need to be addressed through policy and legislative measures, programming, and investments^(23)^. Moreover, many countries have used the NetCode Protocol to assess violations of the WHO Code^(34–36)^.

## Sample selection

Assessing compliance of BMS marketing with relevant regulations using the NetCode Protocol involves interviewing mothers and health professionals and evaluating BMS marketing using samples from three settings: health facilities; retail outlets and product labels; and media.

### Health facilities

Our study selected health facilities that were in the same sample group used in a previous study on ‘Marketing of Breast-Milk Substitutes Thailand’ conducted in 2017^(37)^. These were the main and backup health facilities listed for the previous study^(38)^, and from this we contacted thirty-three main health facilities for their consent to collect data. If they refused, a backup health facility was substituted.

At each health facility, ten mothers of children aged 24 months or less and three health professionals from the children’s vaccination clinic were selected. Mothers were approached on site by asking for their consent to include them in this study. Health professionals were selected by snowball sampling; we contacted a focal point person of each health facility and asked them to select three health professionals who were willing to participate in this study. Furthermore, all educational and informational materials of BMS companies at the facilities were collected.

### Retail outlets

Thirty-three retail outlets in the study were either a small (i.e. not part of a chain) retail shop or pharmacy within a 5-min walk from a selected health facility, while the ten chain stores were selected by purposive sampling. Researchers purchased samples of all available BMS products to review the labels.

### Media

BMS marketing was collected from firstly, the three top-rated TV channels in Thailand (rated by Arianna software used by Nielsen Media Research); and secondly, the internet, on which were the BMS company’s Thai websites and their social media such as Facebook, Instagram, YouTube, Line Official, and also, ten online retailers and ten parenting websites.

## Data collection

### Survey tools, training and administration

The electronic survey tools were developed from the NetCode Protocol^(23)^. These were (1) face-to-face questionnaires for mothers and health professionals; (2) observation and review forms for documenting marketing in health facilities and retail outlets; and (3) recording forms for product labels and BMS marketing in media.

Data were collected from mothers and health professionals, and informational, educational and promotional materials were obtained in health facilities and retail outlets by a team consisting of a key researcher and three data collectors, who attended a 3-d training workshop to learn about WHO Code, the Act and BMS marketing, including the questionnaire and data collection process. These data were collected and recorded on computer tablets through KoboCollect^(39)^, which is a data collection application.

Data from media sources were collected by the media monitoring team. Before data collection, the key researcher trained them in BMS marketing and data collection. These data were recorded on an Excel file.

### Timeline

There were two rounds of data collection from mothers, health professionals, and informational and educational material in health facilities and retail outlets. The first round was conducted between 3 and 19 March 2020, and the second round between 18 June 2020 and 4 August 2020. Data collection from the media was conducted between 1 January and 30 June 2020.

### Measures

Mothers were asked for information about themselves and their children, and their experiences of BMS marketing in the past 6 months. Likewise, health professionals were asked for personal information and their experience in BMS marketing in their health facilities in the past 6 months. We used a semantic differential scale, which is using words rather than numbers to describe respondents’ attitude to products, band, etc.,^(40)^ to measure their opinion on this marketing.

The data collection team observed and recorded or took the materials (if possible) relating to BMS marketing at health facilities and retail outlets. Afterward, the data collection team reviewed the details of the materials such as the type of materials and details of the marketing presented on the materials. For the product labelling survey, we bought all BMS products from retail outlets in Bangkok in March 2020. The label contents were reviewed and recorded on a tablet computer.

The media monitor team collected electronic and digital BMS marketing from TV and the internet between 1 January and 30 June 2020. BMS marketing on TV was monitored for 24 h, over 1 week, every 3 months using Arianna which is a software developed by Nielsen Media Research. Meanwhile, BMS marketing on the internet was collected once a month.

The study asked about infant formula (IF) which is milk for 0–6-month infants, follow-on formula (FF) which is milk for 6–36-month children, growing-up milk (GUM) which is milk for 1–3-year children, any milk intended for children aged 0–36 months (AM), complementary food for children aged less than 6 months (CF < 6), and complementary food for children aged 6–36 months (CF6–36).

Summary details of tools and measures are in supplementary materials (Table S1).

## Data analysis

All data from KoboCollect were exported as Excel files and then cleaned by a researcher. Descriptive statistics were used to present socio-demographic and other characteristics of mothers and health professionals, their experience of BMS marketing, and the opinions of health professionals on BMS marketing at health facilities. It also was applied to characteristics of BMS marketing in health facilities, BMS marketing in retail outlets, product labels and BMS marketing on media.

All BMS marketing in this study, except product labels, was compared with both the WHO Code and the Act by the first author to explore instances of non-compliant BMS marketing with both. Supplementary Table S2 summarises the criteria for compliance under the WHO Code and the Act. Product labels were compared with the WHO Code and with the Ministry of Public Health notifications relating to labels authorised by the Thai Food and Drug Administration (FDA), which is the agency responsible for approving information on labels. There are several regulations for labels, namely the Food Act B.E.2522 and the Notification of the Ministry of Public Health, involved with IF, FF, GUM and CF6-36^(41)^.

The chi-square test was used to assess if opinions on various types of BMS marketing differed between staff who had been trained on the Act and those who had not. A *P*-value ≤ 0·05 indicated significant differences between trained and untrained. This study used STATA version 14.2 for all statistical analyses.

The study received ethics clearance from (1) the Research Ethics Board of the Institute for Development of Human Research Protection, Thailand. (2) Ethics Committee on Human Research of Bangkok Metropolitan Administration, and (3) Science & Medical Delegated Ethical Review Committee (DERC), Australian National University.

## Results

### Characteristics of mothers and breast-milk substitute marketing exposures

The study sample consisted of 330 mothers. Most were 20–29 years old, had graduated from secondary school and had a diploma, were married or lived with their partners and were not in paid employment. In addition, most lived with extended family, of mainly not more than five. They lived in mainly middle-income households, about 15 001–50 000 THB (∼USD 480–1600) per month. At the time of the survey, most children were aged 6 months or older, and most had been born at public health facilities.

Results of BMS marketing exposures found that nearly all (90 %) mothers had experienced at least one type of BMS marketing – the most frequent (82%) was through media. A third of mothers (30%) were exposed to promotions at social groups or events. The third-highest percentage (26%) was of mothers who had received free BMS samples (Table 1).

**Table 1:** Characteristics of mothers and children and their experience in baby food marketing

	*n*	(%)
Characteristics of mothers		
Children’s age (*n* 330)		
Child < 6 months	98	29·70
Child >= 6 months	232	70·30
Mothers’ age (*n* 330)		
< 20 years	31	9·39
20–29 years	179	54·24
30 years and above	120	36·36
Education level (*n* 327)		
Primary school or lower	55	16·82
Secondary school or diploma	224	68·50
Bachelor’s degree or higher	48	14·68
Marital status (*n* 330)		
Live without couple	30	9·09
Live as a couple	300	90·91
Total number of household members (*n* 330)		
1–5 persons	224	67·88
Six persons and above	106	32·12
Type of household (*n* 329)		
Nuclear family	164	49·85
Extended family	165	50·15
CURRENT occupation/employment status of mothers (*n* 327)		
Non-employed/student	169	51·68
Formal work	89	27·22
Informal work	69	21·10
Household monthly incomes (*n* 330)		
0–15 000 THB	95	28·79
15 001–50 000 THB	190	57·58
More than 50 000 THB	45	13·64
Types of a hospital where children were born (*n* 326)		
Public hospitals/clinics	278	85·28
Private hospitals/clinics	48	14·72
Mothers who had an experience in baby food marketing		
Advice about milk formula from others (*n* 329)	74	22·49
Advice about commercially prepared complementary food from others (*n* 330)	68	20·61
Marketing from health facilities (*n* 326)	74	22·70
Marketing from media (*n* 325)	267	82·15
Companies’ social groups and events (*n* 140)	39	30·47
Free baby food sample (*n* 330)	86	26·06
Free coupon relating to baby food products or companies (*n* 329)	24	7·29
Free gift relating to baby food products or companies (*n* 328)	39	11·89

### Breast-milk substitute marketing to health professionals

Data were collected from ninety-nine health professionals using online questionnaires. The sample included five doctors, sixty-four nurses and thirty other health professionals such as pharmacists, social workers and patient assistants. Of these health professionals, 10 % reported having been contacted by BMS companies and 90 % of them were visited directly by companies at health facilities. The most common form of BMS marketing to health professionals (30 %) was providing items for distribution to mothers or introducing themselves when they were a new staff of their companies, or when there was a change in the staff of health professionals (Table 2).

**Table 2:** Characteristics of health professionals and their experience in baby food marketing

	*n*	%
Characteristics of health professionals (*n* 99)		
Position in this health facility		
Doctor	5	5·05
Nurse	64	64·65
Others, for example, pharmacists, social workers and patient assistant	30	30·30
You are familiar with the International Code of Marketing of Breast-Milk Substitutes		
Yes	42	42·42
No/Don’t know	57	57·58
You are familiar with the Control of Marketing of Infant and Young Child Food Act B.E 2560		
Yes	74	74·75
No/don’t know	25	25·25
You have received training on breast-feeding and infant and young child feeding		
Yes	60	60·61
No/don’t know	39	39·39
You have received training on the International Code of Marketing of Breast-Milk Substitutes		
Yes	12	12·12
No/don’t know	87	87·88
You have received training on the Control of Marketing of Infant and Young Child Food Act B.E 2560		
Yes	24	24·24
No/don’t know	75	75·76
In the past 6 months, you have perceived baby food marketing in the health facility		
Yes	10	10·10
No/don’t know	89	89·90
Health professionals who had an experience in baby food marketing (*n* 10)		
Providing items for distribution to mothers	3	30
Providing items for use by your health facility or staff members	2	20
Displaying products or conducting promotional activities in the facility	1	10
Making offers of providing, in future	2	20
Others (introduction themselves and promotion of new products)	3	30

Health professionals reported being aware of WHO Code (42 %), while approximately 74 % said they were familiar with the Act. Just over 60 % of health professionals had been trained in breast-feeding, and infant and young child feeding, but about one-eighth and one-quarter had received training in the WHO Code and the Act, respectively (Table 2).

Table 3 compares health professionals’ opinions on BMS marketing for those trained and not trained in the Act. We found that those who had received training were significantly more likely (*X*^2^ (1, *n* 99) = 5·26, *P* = 0·02) to view ‘companies contacting health professionals to provide items for distribution to mothers and other caregivers of infants and young children’ as inappropriate. The relation between training and adverse opinions on ‘companies providing or offering donations of equipment’ was of borderline significance (*X*^2^ (1, *n* 99) = 3·89, *P* = 0·05)

**Table 3:** Comparison of health professionals’ opinion on baby food marketing between those trained and not trained in the act, number, percentage, andchi-square test of independence

Baby food marketing to health professionals	Training	Opinion				*P*-value
		Non-appropriate		Appropriate		
		*n*	%	*n*	%	
Companies contact health professionals	Yes	23	95·83	1	4·17	0·17
	No	64	85·33	11	14·67	
Companies contact health professionals to provide items for distribution to mothers and other caregivers of infants and young children	Yes	22	91·67	2	8·33	0·02
	No	51	68·00	24	32·00	
Companies contact health professionals to provide items for use by your health facility or staff members	Yes	19	79·17	5	20·83	0·30
	No	51	68·00	24	32·00	
Companies display products or conduct promotional activities in the facility	Yes	21	87·50	2	8·33	0·11
	No	56	74·67	18	24·00	
Companies seek direct contact with mothers	Yes	20	83·33	4	16·67	0·62
	No	59	78·67	16	21·33	
Companies seek direct contact with health professionals	Yes	22	91·67	2	8·33	0·22
	No	60	80·00	14	18·67	
Companies provide or make offer free supplies of any products for 0–36 months	Yes	21	87·50	3	12·50	0·15
	No	55	73·33	20	26·67	
Companies provide or make offer donations of equipment	Yes	21	87·50	3	12·50	0·05
	No	50	66·67	25	33·33	
Companies provide or make offer sponsored events or workshops for health facility staff	Yes	20	83·33	4	16·67	0·22
	No	53	70·67	22	29·33	
Companies provide or make offer payment for or other support to staff to attend events or workshops outside the health facility	Yes	20	83·33	4	16·67	0·10
	No	49	65·33	26	34·67	
Companies provide or make offer the placement of industry-friendly personnel within health organisations	Yes	22	91·67	1	4·17	0·45
	No	68	90·67	7	9·33	

There were missing data for some items, but it was low; 4·17 % and 1·33 % of trained health professionals and non-trained health professionals did not give opinions on ‘displaying products or conducting promotional activities in the facility’, respectively. Missing data for non-trained health professionals’ opinions on ‘seeking direct contact with health professionals’ was 1·33 %, while missing data for trained health professionals’ attitudes towards ‘making an offer of the placement of industry-friendly personnel within health organisations’ was 4·17 %.

### Breast-milk substitutes marketing in health facilities

The presence of promotional, informational or educational materials at the thirty-three health facilities was investigated by data collectors. Of these, thirty-one were public health facilities (twenty-nine primary clinics and two hospitals) of the Bangkok Metropolitan Administration and two were private hospitals.

A third (36 %) of health facilities displayed promotional, informational or educational materials related to BMS products or BMS companies. A total of twenty-two different examples of promotional and informational or educational materials were found at selected health facilities. Most were ‘materials showing company brands or logos’ such as growth charts, followed by informational or educational materials, for example, brochures. This study also found one example of a promotional item which was invitation leaflets for registration to get free product samples (Table 4).

**Table 4:** Characteristics of baby food marketing in health facilities, retail outlets, labels and media

Characteristics of baby food marketing	*n*	%
Health facilities (*n* 23)		
Type of promotional and informational materials at health facilities		
Equipment showing company brands or logos	16	69·57
Promotional materials	1	4·35
Informational or educational materials	6	26·09
Target audience		
General public	23	100·00
Health workers (i.e. has wording that states that the material is intended for healthcare workers only)	0	0·00
There are brand names/logos shown		
Yes	15	65·22
No	8	34·78
The materials include the appropriate age of introduction		
Yes	2	8·70
No	21	91·30
Retail outlets (*n* 265)		
Baby food promotion		
Price related (e.g. coupon/stamps, discounts and special discount sales)	155	58·49
Displays (e.g. brand shelf, special displays, shop window, posters/banners, shelf tag/talkers and product launch)	60	22·64
Information materials (e.g. pamphlets, booklets and leaflets)	16	6·04
Free gifts	30	11·32
Product samples	0	0·00
Company representative	1	0·38
Others	3	1·13
Target audience		
General public	265	100·00
Health workers (i.e. has wording that states that the material is intended for healthcare workers only)	0	0·00
There are brand names/logos shown		
Yes	197	74·34
No	68	25·66
The materials include the appropriate age of introduction		
Yes	207	78·11
No	58	21·89
Labels (*n* 213)		
Type of products		
Infant formula (IF)	29	13·62
Follow-up/follow-on formula (FF)	17	7·98
Growing-up milk/toddler milk (GUM)	36	16·9
Any other milk intended for children aged 0–36 months (AM)	2	0·94
Commercial complementary food or drinks intended for infants less than 6 months of age (CF < 6)	0	0·00
Commercial complementary food or drinks intended for infants or young children between 6 and 36 months (CF6–36)	129	60·56
Criteria for all labels		
Product information is printed on the container or a well-attached label	213	100
The language used on the product label is appropriate for the country in which the product is sold	213	100
Contains any nutrition and/or health claims	93	43·66
Conveys an endorsement by a health worker or health professional body	17	7·98
Includes the recommended or appropriate age of introduction	182	85·45
Includes the contact details of companies.	192	90·14
Contains promotional devices to induce sales of the company’s products under the scope	14	6·57
Includes a list of the ingredients	213	100
Displays nutritional composition of the product	206	96·71
Contains storage instructions	176	82·63
Contains batch number	72	33·8
Shows the date before which the product should be consumed (expiration date)	213	100
Additional criteria for baby milk products		
Includes the words ‘Important Notice; or their equivalent	43	51·19
Includes a statement on the superiority of breast-feeding	47	55·95
Contains text or images that may idealise the use of breast-milk substitutes	38	45·24
Media: TV (*n* 14)		
Type of baby food marketing		
Advertisement	14	100·00
Product types mentioned in the promotion		
GUM	14	100·00
Media: the internet		
Media source (*n* 931)		
Manufacturer of baby food products	842	90·44
Mothers’ magazines/online forums	31	3·33
Online retailers	57	6·12
Influencers	1	0·11
Type of channel (*n* 931)		
Website	156	16·76
Facebook	443	47·58
Twitter	0	0·00
YouTube	55	5·91
Instagram	106	11·39
Other (line official)	171	18·37
Type of baby food marketing (*n* 931)		
Advertisement	206	22·13
Information note	188	20·19
Interview	5	0·54
News report	10	1·07
Opinion/analysis/debate	16	1·72
Viral marketing encouraging mothers to contact their peers about specific products or brand	14	1·50
Sweepstakes and promotions	13	1·40
Club memberships	37	3·97
Incentives for products purchase	121	13·00
Others (price related, product review, Facebook live and providing gifts or items)	321	34·48
Product types mentioned in the promotion (*n* 940)		
Infant formula (IF)	8	0·85
Follow-on formula (FF)	6	0·64
GUM	467	49·68
Commercial complementary food or drinks intended for infants or young children between 6 and 36 months (CF6–36)	292	31·06
Not a specific product	167	17·77

In health facilities, 56·5 % of all promotional and informational materials were in Thai and English languages. All of them ostensibly targeted the public and were produced or created by BMS manufacturers. More than 50 % of the materials showed brand names or logos, but most (73 %) did not specify product types. Three out of the twenty-three promotional and informational materials made claims that purported health or nutrition benefits of using the products, by conveying wording or messages such as ‘health’, ‘nutritious’ and ‘enhances child growth’. Moreover, 26 % of all materials aimed to educate the recipient about infant and young child feeding or provide advice about feeding (Table 4).

### Breast-milk substitute marketing at retail outlets

The study included thirty-six retail outlets; twenty-six small retail outlets or pharmacies and ten large chain supermarket stores. About 46 % of small outlets or pharmacies showed BMS marketing, while eight out of ten chain stores had such marketing. The total number of BMS marketing items at retail outlets was 265. Mainly, BMS marketing was ‘price-related’ (Table 4).

All BMS marketing items at retail outlets targeted the public and were produced or created either by retailers or BMS companies. The number of marketing items showing brand names or logos was 197, and 207 out of 265 marketing items presented an appropriate age of introduction. GUM was the product that showed most brand names or logos.

Marketing of products (6·79 %) used words like ‘new’, ‘improve’, ‘similar to breast milk’, ‘health’, etc., to suggest the benefits. Moreover, 1·54 % of BMS marketing materials educated the recipient about infant and young child feeding or provided feeding advice on topics such as ‘maternal nutrition’ or how to ‘promote bottle feeding’. 80·75 % of materials used Thai and English languages, and 84·15 % of them were created by retailers. (Table 4).

### Breast-milk substitutes product labels

In total, 213 BMS product labels were collected. 60·56 % of labels were for CF6–36. Approximately 43 % of all product labels presented nutrition or health claims. About half of all formula labels showed the ‘Important Notice’, the requirement of the FDA to indicate to consumers the importance of breast-feeding or how to use the products. All IF and about 76 % of FF labels included the important notice, while none of the GUM products presented this notice on their labels. All IF and FF showed included a statement on the superiority of breast-feeding, but no GUM products had the statement (Table 4).

None of the formula labels contained text or images that discouraged or undermine breast-feeding. However, about 9 % contained information that implies or creates a belief that BMS products are equivalent or superior to breast milk. As well, 58·33 % and 63·1 % of formula labels contained a statement about the need for health worker advice on the proper use of the product and a warning about the health hazards of inappropriate preparation and usage, respectively.

Furthermore, only 20 % of powdered milk products warned that the product may contain pathogenic micro-organisms, although all presented clear graphic instructions illustrating the method of preparation. About 82 % of these labels showed the use of hygienic practices, while all of them show the need to boil water and sterilise utensils. More than half the formula labels displayed the necessity for a powdered formula to be prepared one feed at a time (52·63 %) and leftovers to be discarded immediately (78·95 %). About 18 % of these formula labels showed the need to cool the formula before feeding if using hot water for reconstitution.

No commercially prepared complementary food had a statement on the importance of continuing breast-feeding for at least 2 years. However, 62 % of all complementary food products included the statement that ‘the product should not be given to infants under 6 months’. None of them included ‘text or images suggesting that the product should be given to infants under 6 months’ or ‘text or images that may discourage or undermine breastfeeding’ or ‘information that implies or creates a belief that complementary foods are equivalent or superior to breast milk’ or the suggestion to use this product with a bottle. Furthermore, none of the commercially prepared complementary food labels were similar to the formula produced by the same manufacturer.

### Breast-milk substitute marketing in electronic media

In total, 14 different BMS advertisements were collected from three TV channels, alongside 931 instances of online marketing. All BMS marketing on TV were advertisements for GUM in Thai and English, while about 86 % of them showed the purported benefits of using the product. (Table 4).

In the same period, the internet review revealed various types of BMS marketing. Approximately 34 % of all BMS marketed on the internet used ‘other’ marketing techniques such as reviews of products by mothers or influencers, Facebook live events with health professionals and providing items such as gifts to mothers. We collected 842 examples of BMS marketing instances from websites and the social media of BMS companies which was the major source of digital marketing. In addition, Facebook had the highest number of marketing instances at 443. Approximately 99 % of marketing on the internet was only in Thai and 17 % of these advertisements contained wording or messages claiming health or nutrition benefits of the products (Table 4).

### Compliance of breast-milk substitute marketing with the WHO Code and the Act

Table 5 presents, in summary, the percentage of all instances of non-compliance by BMS marketing with the WHO Code. In this study, there were 2856 instances of BMS marketing that did not comply with the WHO Code. BMS marketing through media comprised the highest percentage of WHO Code violations (accounting for 35·64 % of all non-compliance instances), followed by labelling of products and marketing to mothers, respectively. The most predominant violation of BMS marketing on electronic media targeting mothers was TV advertisements.

**Table 5:** The percentage of instances of baby food marketing identified as non-compliant with WHO Code

Channel of marketing	Percentage of instances of non-compliant baby food marketing with WHO Code by product types							
	IF	FF	GUM	AM	CF < 6	CF6–36	Non-specific	Total
Mothers	5·57	2·28	8·40	5·18	0·32	1·05	1·72	24·51
Health professionals	0·00	0·00	0·00	0·07	0·00	0·00	0·00	0·07
Health facilities	0·00	0·00	0·11	0·14	0·00	0·00	0·35	0·60
Retail outlets	0·60	0·14	7·42	0·77	0·00	0·18	0·18	9·28
Media	0·49	0·42	18·49	0·00	0·00	10·40	5·85	35·64
Labels	4·13	2·45	10·47	0·46	0·00	12·39	0·00	29·90
Total	10·78	5·29	44·89	6·62	0·32	24·02	8·09	100·00

IF, infant formula; FF, follow-on formula; GUM, growing-up milk; AM, any milk intended for children aged 0–36 months; CF < 6, complementary food for children aged less than 6 months; CF6–36, complementary food for children aged 6–36 months; non-specific, a promotion did not show product types or appropriate age of introduction of the products.

Marketing of GUM products violated the WHO Code more frequently than others, particularly by advertising on media, and through discount sales and special displays at retail outlets. Marketing of complementary food products for 6–36-month children was the second most likely to fail to comply with WHO Code, for example, the products lacked the statement ‘the product should not be given to infants under 6 months’ or did not show storage instructions (Table 5).

Table 6 presents the percentage of all BMS marketing, that is, non-compliant with the Thai Act and labelling regulations of the FDA. There were 227 instances of BMS marketing that was non-compliant with the Act. Most were examples of BMS marketing to mothers (*n* 189, 83·26 % of all non-compliant instances), following by marketing through retail outlets (*n* 21, 9·25 %) and media (*n* 17, 7·49 %). The most frequent violation was for IF. Examples of violations of marketing to mothers occurred in advertising and by offering IF to mothers. Ninety-six labels did not follow FDA regulation with most (*n* 92) being for CF-36. Labels did not reveal the composition/analysis of the product, or the storage conditions required.

**Table 6:** The percentage of instances of baby food marketing identified as non-compliant with the Act and FDA regulations

Regulation	Channel of marketing	Percentage of instances of non-compliant baby food marketing with Thai regulations by product types				
		IF	FF	CF < 6	CF6–36	Total
The Act	Mothers	54·19	26·87	2·20	N/A	83·26
	Health professionals	0·00	0·00	0·00	N/A	0·00
	Health facilities	0·00	0·00	0·00	N/A	0·00
	Retail outlets	7·49	1·76	0·00	N/A	9·25
	Medias	3·96	3·52	0·00	N/A	7·49
	Total	65·64	32·16	2·20	N/A	100·00
FDA	Labels	0·00	4·17	N/A	95·83	100·00

IF, infant formula; FF, follow-on formula; CF < 6, complementary food for children aged less than 6 months; CF6–36, complementary food for children aged 6–36 months; N/A, non-applicable.

## Discussion

In this study, we examined the types, prevalence and compliance of BMS marketing in Thailand with the Act and the WHO Code. The findings show considerable BMS marketing to mothers and health professionals in Bangkok, Thailand, that did not comply with the WHO Code and related international guidance on inappropriate marketing^(42)^. Furthermore, BMS companies promoted their product to mothers and the public through health facilities, retail outlets, product labelling and electronic media. Overall, the survey results showed BMS marketing in Thailand did not comply with either the Act or the WHO Code, despite several years passing since the enactment of Thailand’s new national law.

The results revealed that most mothers were exposed to at least one type of BMS marketing, with the most frequent marketing channel being the electronic media. These findings reflect previous studies in Indonesia and Mexico, where the results showed most mothers saw the promotion of BMS in media^(22,43)^.

Most health professionals reported they had been contacted by companies or were visited by representatives of BMS companies at health facilities. Companies mainly contacted health professionals to provide items for them to distribute to mothers. A recent study in Ecuador found similarly that about 90 % of selected health professionals were contacted by BMS companies^(44)^.

Our results revealed that only a minority of selected health professionals were trained on the Act or the WHO Code. In Thailand, the Department of Health had a plan to train 25 % of all responsible people by 2020 and intended that all responsible people will be trained about the Act by 2022^(26)^. Furthermore, findings illustrated lack of training was associated with health professionals’ having positive opinions regarding receiving items from the companies for distribution to mothers.

Most BMS marketing in retail shops was price-related which was the same result as a similar previous study in Bangkok, which found that the most frequent form of promotions in retail shops was price discounts (39·1 %). Furthermore, not all of the reviewed labels of GUM presented the Required Notice, which is an informational statement for consumers, for example, ‘Breastfeeding is most superior for infants because of its complete nutrition value’ and ‘Modified milk for infants should be used on the advice of a doctor, nurse or nutritionist’ because products’ labels are controlled by FDA^(41)^. FDA has a specific requirement for Notification for IF and FF^(45,46)^, but there is no such Notification requirement for GUM. Therefore, labels of GUM products are regulated as other milk products despite the importance of protecting breast-feeding for children aged 12–36 months and good nutrition in early childhood.

BMS marketing in the media was the main form of violation of the WHO Code, and most of these violations were TV advertisements. GUM was the product that most frequently violated the WHO Code. These results were similar to the study by the Access to Nutrition Foundation in 2017^(37)^. By contrast, the most common violation of the Thai Act was BMS marketing to mothers, and IF product marketing was the most common violation of the Act. Recently, a review of the scope and impact of digital marketing of BMS has proposed new national and international initiatives to address this type of marketing^(47)^. This issue is being reviewed by WHO to develop new guidance on restricting digital marketing^(48)^.

A key reason for discrepant results on non-compliance with WHO Code^(49)^ and the Act^(27)^ is because the Act and the WHO Code are not exactly consistent with each other, even though the Act was developed based on WHO Code. The scope of products covered by the Act does not include feeding bottles and teats, or complementary foods for young children, while the WHO Code covers such products. Additionally, GUM are not covered by the Act, with the consequence that the restraints on marketing through cross-promotion intended by the WHO Code are still not enacted in Thai law. This has at least partially resulted from the interference of BMS companies in the legislation of the Act, at both national and international levels^(28,29)^.

This study has some limitations. First, it collected data in Bangkok only, since the NetCode Protocol for the periodic^(23)^ study recommends that monitoring should be conducted in the capital or largest city of the country, where BMS marketing is more common. Therefore, BMS marketing trends that may be specific to smaller cities or other provinces were excluded from this study. Company and company product lines were also not identified in this study.

Second, as in the NetCode Protocol, mothers and health professionals were asked to recall their experiences in BMS marketing in the past 6 months, so their memory of BMS marketing may not be accurate. However, this time period is considered appropriate for recalling salient advertising.

Third, the emergence of the COVID-19 pandemic in 2020 led to changes in circumstances and data collection times. The COVID-19 pandemic may have contributed to changes in marketing techniques for BMS^(50)^. Moreover, in Thailand, there was a donation of formula milk through government organisations such as the Ministry of Social Development and Human Security to mothers or to the Provincial Public Health Office for mothers who were considered in need^(51)^. This was not in compliance with the Operational Guidance on Infant and Young Child Feeding in Emergencies which states: ‘Do not donate or accept donations of BMS, other milk products or feeding equipment (including bottles, teats and breast pumps) in emergencies’^(52)^. During the COVID-19 pandemic, exclusive breast-feeding was shown to decline in some countries, including Italy and Thailand^(53,54)^. Hence, the changed circumstances and data collection timing is a limitation of the study as the pandemic could not be anticipated. However, collecting data in the early and later months of 2020 may support future research on whether BMS marketing and compliance changed during this period in Thailand.

Monitoring compliance requires an evaluation programme to find evidence for modification of ineffective policies and to make enforcement more effective. It is important to reflect on why participants in the industry do not comply with either Thai law or the WHO Code, despite the Department of Health having two surveillance systems to monitor violations of the Act. The active monitoring system involves government officials who are authorised by Department of Health to monitor and report violations at target settings such as health facilities or retail outlets. The passive monitoring system relies on members of the public reporting violations to authorised government officials^(26)^. As well, the Act has sanctions^(27)^.

It has been suggested that to improve regulatory compliance, policymakers should not stop at regulation, but need to also include communication strategies such as information campaigns to ensure that target groups, including the public, are aware of the goals of regulation and understand how to comply with the law^(55)^. This implies that improving the regulation of marketing of BMS requires not only regular monitoring of levels of compliance with the Act and its enforcement but also communication strategies to provide knowledge and information about the Act to stakeholders such as health professionals and civil society organisations which help motivate and support compliance.

Future research needs to extend the study area to other provinces including the smaller ones to cover all regions of Thailand. There should be a regular assessment of compliance of all BMS marketing with the Act including online, and in addition, BMS marketing should be studied to identify trends by company, company product line and location.

## Conclusions and policy implications

This study demonstrates that improved policy implementation, monitoring and enforcement are needed to improve BMS marketing compliance in Thailand. The policy recommendations are as follows. First, the Ministry of Public Health should regularly and comprehensively assess the compliance of BMS marketing with the Act and improve and enforce the law and penalties without influence from BMS companies. Second, the Act should be revised to cover GUM and to align with the WHO Code, including recent guidance on ending the practice of cross-promotion, and provide upcoming technical guidance on digital marketing. Third, health professionals and relevant health organisations involved with mothers and children might be required to follow the Act and WHO Code strictly to ensure that health professionals and health facilities cannot be exploited as channels to promote BMS products. Furthermore, all health professionals should receive training and ongoing communications about the Act and the WHO Code. Lastly, the monitoring and surveillance of BMS marketing in digital media should be strengthened, since it is extensively practiced using a variety of techniques and is now the main source of marketing exposure. Communications campaigns could raise awareness and public support for improved compliance and enforcement.

## Supplementary material

For supplementary material accompanying this paper visit https://doi.org/10.1017/S1368980022001446. click here to view supplementary material
