# Supplementary material for: Regulation of baby food marketing in Thailand: a NetCode analysis
Source: Public Health Nutr. 2022 Jun 23;25(10):2680–92. doi: 10.1017/S1368980022001446 (PMC9991832; doi:10.1017/S1368980022001446)
Supplement: Supplementary file 1 [file S1368980022001446sup001.docx]

**Supplementary materials**

**Table S1 Summary of data collection methods**

| **Setting** | **Sample group** | **Selection** | **Type of tools** | **Question topics** | **Data collection and measures** |
| --- | --- | --- | --- | --- | --- |
| Health facilities | 330 mothers of children less than or equal to 24 months at the children’s vaccination clinic. | - 10 mothers from each selected health facility. - Researchers approached eligible mothers and asked for their consent to include them in this study. | The electronic questionnaire was administered face-to-face | 1. Mothers’ information such as age, education, marital status, number of household members, household income, and occupation. 2. Children’s information such as place of birth, age 3. Experience in BMS marketing in the past six months  - Advice from other people - Promotion through media - Social group and event - Receiving items such as product samples, coupons, and gifts | - Data was collected by interviewing mothers. - Data was analysed by descriptive statistics. |
|  | 99 health professionals working in selected health facilities | - Researchers asked a focal point person of each health to select three health professionals from their health facility. - Selected health professionals gave their consent to collect their data | The electronic questionnaire was administered face-to-face | 1. Health professionals’ information 2. Experience in BMS marketing in the past six months, and opinion on such marketing.  - Contacted by BMS companies to health professionals - Contacted by BMS companies to provide items for distribution to mothers - Providing items to health facilities - BMS companies looked for mothers’ contact details - BMS companies looked for health professionals’ contact - BMS companies gave free samples - BMS companies support health professionals to attend conferences - BMS companies made an offer of providing something in the future | - Data was collected by interviewing health professionals. - Data was analysed by descriptive statistics. |
|  | 33 health facilities | The first author listed health facilities from the study of Access to Nutrition Foundation. Then, the first author contacted the health facilities to ask them for consent to collect data. | Electronic observation and review form | 1. Types of informational or educational materials 2. Materials appear to be intended to be given to patients to take home 3. Target group 4. Baby food companies or their brand which appear on materials 5. Product types are mentioned in the materials 6. The material includes the appropriate age of introduction 7. Language 8. Messages about the benefits of using the product 9. Messages about infant and young child feeding 10. Messages about bottle-feeding 11. Pictures that may idealize the use of breast-milk substitutes or discourage/ undermine breastfeeding | - Data was collected by observation and review of promotional and educational materials in health facilities. - Data was analysed by descriptive statistics. |
| Retail outlets | 43 retail outlets (10 big shops and 33 small shops) | - One small retail outlet selected from retail outlets or pharmacies which were far from the selected health facility around a 5-minute walk. - 10 big shops were selected from chain stores in Thailand. | Electronic observation and review form | - Types of promotion - Type of product that is promoted - Target group - Baby food companies or their brand which appear on materials - Product types are mentioned in the materials - The material includes the appropriate age of introduction - Language - Messages about the benefits of using the product - Messages about infant and young child feeding - Messages about bottle-feeding - pictures that may idealize the use of breast-milk substitutes or discourage/ undermine breastfeeding | - Data was collected by observation and review of BMS marketing in retail outlets. - Data was analysed by descriptive statistics. |
|  | Product labelling | All labels of formula milk and complementary food for 0-36-month children were included. | Electronic record form | 1. All products  - Company’s name - Product name - Type of product - Language - Nutrition and health claim - Ingredients  1. Formula milk products  - Text or images - Statement or information - Warning of contamination - Instruction of preparation  1. Complementary food products  - Text or images - Statement - Similarity with Formula milk products | - Data was collected by reviewing product labels. - Data was analysed by descriptive statistics. |
| Media | Media (TV and the internet) | - Three top-rated TV channels - All baby food company websites and social media - 10 online retail outlets - 10 parenting websites | Electronic record form | 1. All media  - Type of promotion - Type of product - Language - Messages about benefits of products  1. TV  - -Channel - Transmission time  1. The internet  - Media source - Type of channel such as website, Facebook | - Data was collected by monitoring baby food marketing from the media. - Data was analysed by descriptive statistics. |

**Table S2 Criteria for compliance of baby food marketing with WHO Code and Thai regulations**

| **Compliance criteria** | **WHO Code** | **The Act/FDA** |
| --- | --- | --- |
| ***Informational and educational materials*** | | |
| Requirement of informational and educational materials for **all baby food products** |  |  |
| - The benefits and superiority of breastfeeding; | / |  |
| - Maternal nutrition, and the preparation for and maintenance of breastfeeding; | / |  |
| - The negative effect on breastfeeding of introducing partial bottle-feeding; | / |  |
| - The difficulty of reversing the decision not to breast-feed; | / |  |
| Requirement of informational and educational materials for **infant formula** |  |  |
| - The social and financial implications of its use | / | / |
| - The health hazards of inappropriate foods or feeding methods; | / |  |
| - The health hazards of unnecessary or improper use of infant formula and other breast-milk substitutes. | / |  |
| - Such materials should not use any pictures or text which may idealize the use of breast-milk substitutes. | / |  |
| ***Promotion to the general public*** | | |
| No advertising or other form of promotion to the general public of baby food products. | / | / |
| No providing free samples of baby food products from baby food companies. | / | / |
| No point-of-sale promotion either online shops or retail outlets. | / | / |
| No gifts and coupons from baby food companies | / | / |
| Baby food companies should not direct or indirect contact with mothers. | / | / |
| ***Promotion in health care systems*** | | |
| No advice on baby food products from a health professional. | / |  |
| No distribution of items relating to baby food products to mothers via health professionals. | / |  |
| Promotional material in health facilities should not refer to baby food products. | / |  |
| Equipment donated to health facilities should not refer to baby food products | / | / |
| ***Promotion to Health workers*** | | |
| Product information for health professionals is restricted to scientific and factual matters. | / | / |
| No sponsorship, financial support, or materials to health workers. | / | / |
| Health workers should not give samples of baby food products to mothers | / |  |
| ***Labelling*** | | |
| Requirement of labelling for **all baby food** |  |  |
| - Product information is printed on the container or a well-attached label | / |  |
| - The language used on the product label is appropriate for the country in which the product is sold | / |  |
| - No text or images that may idealize the use of breast-milk substitutes | / |  |
| - Include the appropriate age of introduction of the food | / |  |
| - No text or images that may discourage or undermine breastfeeding | / |  |
| - No information that implies or creates a belief that breast-milk substitute products are equivalent or superior to breast milk | / |  |
| - Ingredients | / | / |
| - Composition/analysis of the product | / | / |
| - Storage conditions | / | / |
| - Batch number | / |  |
| - Expiration date | / | / |
| - Nutrition and/or health claims | / |  |
| - Warning that powdered baby milk products may contain pathogenic microorganisms | / |  |
| - Conveys an endorsement by a health worker or health professional body | / |  |
| Requirement of labelling for **milk formula** |  |  |
| - The words "Important Notice" or their equivalent; | / | / |
| - A statement of the superiority of breastfeeding; | / | / |
| - A statement that the product should be used only on the advice of a health worker as to the need for its use and the proper method of use; | / | / |
| - Instructions for appropriate preparation, and a warning against the health hazards of inappropriate preparation. | / | / |
| - The label shows clear graphic instructions illustrating the method of preparation | / |  |
| - Instructions show the use of hygienic practices, e.g., clean hands, preparation surfaces | / |  |
| - Instructions show the need to boil water and sterilize utensils | / |  |
| - Instructions show the necessity for powdered milk formula to be prepared one feed at a time | / |  |
| - Instructions show the need to cool the formula before feeding if using hot water for reconstitution | / |  |
| - Instructions show that left-overs of the product need to be discarded immediately | / |  |
| Requirement of labelling for **complementary food** |  |  |
| - Statement on the importance of continuing breastfeeding for at least 2 years | / |  |
| - Statement that the product should not be given to infants under 6 months | / | / |
| - Recommend or promote bottle feeding | / |  |

**List of abbreviations**

| The Act | the Control of Marketing of Infant and Young Child Food Act B.E. 2560 |
| --- | --- |
| AM | Any milk intended for children aged 0-36 months |
| BMS | Breast-milk substitute |
| CF<6 | Complementary food for children aged less than 6 months |
| CF6-36 | Complementary food for children aged 6-36 months |
| CMF | Commercial milk formulas |
| The Code | the International Code of Marketing Promotion of Breast-milk Substitutes |
| DOH | Department of Health |
| FDA | Thai Food and Drug Administration |
| FF | Follow-on formula |
| GUM | Growing-up milk |
| IF | Infant formula |
| THB | Thai Baht |
| USD | United States Dollar |
| WHA | World Health Assembly |
| WHO | World Health Organization |
